# Mind the Gap: How Interspecies Variability in IgG and Its Receptors May Complicate Comparisons of Human and Non-human Primate Effector Function

**DOI:** 10.3389/fimmu.2019.00697

**Published:** 2019-04-08

**Authors:** Andrew R. Crowley, Margaret E. Ackerman

**Affiliations:** ^1^Molecular and Cellular Biology Program, Dartmouth College, Hanover, NH, United States; ^2^Thayer School of Engineering, Dartmouth College, Hanover, NH, United States

**Keywords:** non-human primate, rhesus, cynomolgus, HIV, SIV, IgG, Fcγ receptor, neonatal Fc receptor

## Abstract

The field of HIV research relies heavily on non-human primates, particularly the members of the macaque genus, as models for the evaluation of candidate vaccines and monoclonal antibodies. A growing body of research suggests that successful protection of humans will not solely rely on the neutralization activity of an antibody's antigen binding fragment. Rather, immunological effector functions prompted by the interaction of the immunoglobulin G constant region and its cognate Fc receptors help contribute to favorable outcomes. Inherent differences in the sequences, expression, and activities of human and non-human primate antibody receptors and immunoglobulins have the potential to produce disparate results in the observations made in studies conducted in differing species. Having a more complete understanding of these differences, however, should permit the more fluent translation of observations between model organisms and the clinic. Here we present a guide to such translations that encompasses not only what is presently known regarding the affinity of the receptor-ligand interactions but also the influence of expression patterns and allelic variation, with a focus on insights gained from use of this model in HIV vaccines and passive antibody therapy and treatment.

## Introduction

Advances in the treatment and prevention of HIV have relied heavily on the macaque animal model. Infection of animals with simian immunodeficiency virus (SIV) or chimeric simianized human immunodeficiency virus (SHIV) allows researchers to both model transmission and induce in a variety of macaque species a disease state characterized by sequelae closely resembling those found over the course of human HIV infection (1–5), against which therapeutic interventions can be compared. The similarities of the macaque model to humans has permitted the screening of drug candidates prior to elaborate and expensive human trials, which has contributed to the development of lifesaving drugs such as tenofovir (6, 7). In addition to its successes in the development of small molecule inhibitors, work in macaques has demonstrated that vaccines and antibodies are capable of mediating protection against SIV/SHIV infection, offering important insights into the characteristics of effective immunological responses that serve as a model for vaccine research and development.

However, the high-profile failures of all but one of the vaccine candidates tested in humans to protect against HIV-1 infection also suggest the limitations of the macaque model. For all of the beneficial contributions the model has made to the understanding and treatment of HIV, discrepancies between outcomes in macaque studies and in the clinic suggest that a better understanding of how human and macaque immunobiology differ may be necessary in order to more reliably draw translatable conclusions from animal studies. Fundamental differences between humans and macaques, here focusing particularly in the area of humoral immune responses, mean that in some cases, the result of a vaccination study or a passive transfer experiment conducted in macaques may not be recapitulated if attempted in humans. Promisingly, with a more complete understanding of this interspecies diversity, it should be possible to better translate observations likely to be meaningful to human protection and therapy. While numerous Fc receptors for IgG exist, many of these, including FcRL5, mucins, and TRIM21 (8–10), have yet to be evaluated thoroughly in the macaque model. However, recent work has greatly enhanced our knowledge of IgG, FcγR, and FcRn biology in rhesus macaques. This review summarizes the current understanding of what differences exist in the setting of receptor and antibody interactions between humans and non-human primates, with a particular emphasis on those facets that have the potential to affect the evaluation of candidate vaccines and antibody-based strategies for the prevention or treatment of HIV-1 infection in humans.

## Antibodies in HIV Prevention and Therapy

A wealth of data from non-human primate studies has demonstrated that monoclonal antibodies can provide sterilizing protection from viral challenge (11–13); additionally, because humoral immune responses are often correlates of vaccine-mediated protection, it has become clear that closer inspection of immunoglobulin biology in macaques has the potential to significantly advance research and development of vaccines and therapeutic antibodies. This potential was made all the more apparent following the conclusion of the single successful human vaccine trial to date, the RV144 trial conducted in Thailand, in which a moderate degree of protection was observed (14). A subsequent study of vaccine-induced immune responses identified binding, rather than broadly neutralizing, antibodies directed against the envelope glycoprotein as a correlate of reduced risk of infection (15). Furthermore, additional correlates analysis suggested that binding antibody responses with certain characteristics, such as a bias toward IgG3, away from IgA, and with antibody-dependent cell-mediated cytotoxicity (ADCC), and complement cascade-initiating activity were associated with reduced risk (15–18). These findings reinforce those of numerous other studies in both humans and macaques (summarized in Table 1) that have concluded that innate immune effector functions, such as ADCC, correlate with and may directly contribute to improved outcomes at all stages of HIV infection, from potentially blocking acquisition of the virus altogether to maintaining a lower viral load and delaying the onset of AIDS.

**Table 1:** A partial summary of human and NHP studies that investigated the role of antiviral antibody effector functions in the context of (S)HIV infection.

**Year**	**Species**	**Setting**	**Relevant findings, quoted**	**References**
**Fc RECEPTOR-MEDIATED EFFECTOR FUNCTION IS ASSOCIATED WITH REDUCED ACQUISITION OF HIV-1/SIV/SHIV**				
2018	*M. mulatta*	Vaccination	Reduced risk of infection was associated with IgG-driven antibody-dependent monocyte-mediated phagocytosis in the [intramuscular] vaccinees, but with vaccine-elicited IgA-driven neutrophil-mediated phagocytosis in [aerosol]-immunized animals	(19)
2017	*M. mulatta*	Vaccination	Systems serology of the antibody responses identifies plasma antibody binding to HIV-infected cells, peak ADCC antibody titres, NK cell-mediated ADCC and antibody-mediated activation of MIP-1b in NK cells as the four immunological parameters that best predict decreased infection risk	(20)
2015	*M. mulatta*	Vaccination	Protective efficacy correlated with the functionality of Env-specific antibody responses	(21)
			These data demonstrate robust protection by Ad/Env vaccines against acquisition of neutralization-resistant virus challenge in rhesus monkeys	
2015	*M. mulatta*	Vaccination	Protection correlated with antibody-dependent cellular cytotoxicity specific for CD4-induced epitopes, provided that the concurrent antivaccine T-cell responses were minimal. Protection was lost in instances when T-cell responses were high or when the requisite antibody titers had declined	(22)
2014	*H. sapiens*	Vaccination	These data suggest that subclass selection differences associated with coordinated humoral functional responses targeting strain-specific protective V2 loop epitopes may underlie differences in vaccine efficacy observed	(16)
2013	*M. mulatta*	Vaccination	Protection against acquisition of infection correlated with vaccine-elicited binding, neutralizing, and functional non-neutralizing antibodies	(23)
2012	*M. mulatta*	Vaccination	Measures of ADCC activity were higher among the SIVΔnef-inoculated macaques that remained uninfected than among those that became infected	(24)
2012	*H. sapiens*	Vaccination	The binding of IgG antibodies to variable regions 1 and 2 (V1V2) of HIV envelope proteins (Env) correlated inversely with the rate of HIV-1 infection	(15)
2011	*M. mulatta*	Vaccination	All protected animals showed gp41-specific vaginal IgAs with HIV-1 transcytosis-blocking properties and vaginal IgGs with neutralizing and/or [ADCC] activities	(25)
			Plasma IgGs totally lacked virus-neutralizing activity	
2007	*H. sapiens*	Vaccination	The level of vaccine-induced ADCVI activity correlated inversely with the rate of acquiring HIV infection	(26)
			ADCVI correlated poorly with neutralizing or CD4-gp120-blocking Ab activity	
			degree to which the ADCVI Ab response predicted the rate of infection was influenced by polymorphisms at the FcgR2a and FcgR3a gene loci	
2007	*M. mulatta*	Passive transfer	There is a dramatic decrease in the ability of a broadly neutralizing antibody to protect macaques against SHIV challenge when Fc receptor and complement-binding activities are engineered out of the antibody	(27)
			No loss of antibody protective activity is associated with the elimination of complement binding alone	
1998	*M. mulatta*	Passive transfer	SIV hyperimmune sera given subcutaneously prior to oral SIV inoculation protected 6 newborns against infection	(28)
			SIV hyperimmune sera was given… 3 weeks after oral SIV inoculation, viremia was not reduced	
**Fc RECEPTOR-MEDIATED EFFECTOR FUNCTION IS ASSOCIATED WITH REDUCED VIREMIA AND/OR HIGHER CD4**^**+**^ **T CELL COUNTS**				
2018	*M. mulatta*	Challenge model	There was a significant reduction in the seeding of virus to the lymph nodes and a decrease in plasma viremia in the HIV antibody-infused macaques compared with the control antibody-infused animals	(29)
2016	*H. sapiens*	Infected patients	[elite controllers] demonstrated polyfunctional humoral immune responses able to coordinately recruit ADCC, other NK functions, monocyte and neutrophil phagocytosis, and complement	(30)
2016	*M. mulatta*	Vaccination	gp140-specific IgG3 Abs of females but not males were correlated with [ADCC] against gp120 targets (*p* = 0.026) and with [ADCP] (*p* = 0.010)	(31)
			IgG3 Ab of females but not males also correlated with decreased peak viremia (*p* = 0.028)	
2015	*M. mulatta*	Passive transfer	Passive infusion of each of the three antibodies significantly reduced the number of [transmitted/founder] genomes	(32)
2011	*M. mulatta*	Vaccination	Pre- and post-challenge memory B cells were correlated with functional antibody responses including [ADCC], [ADCVI], and transcytosis inhibition	(33)
			Post-challenge, Env-specific IgG and IgA memory B cells were correlated with reduced chronic viremia	
2011	*H. sapiens*	Infected patients	ADCC responses to whole gp140 Env protein were strongly associated with a slower decline in CD4 T cell loss	(34)
2013	*H. sapiens*	Infected patients	Found significantly higher levels of ADCC antibodies in controllers verses viremic subjects (*p* = 0.017)	(35)
2010	*M. mulatta*	Vaccination	Both ADCVI and percent ADCC killing prechallenge were significantly correlated with reduced acute viremia	(36)
			[percent ADCC killing prechallenge], as well as post-challenge ADCVI and ADCC, was also significantly correlated with reduced chronic viremia	
2009	*M. mulatta*	Vaccination	The higher ADCC and ADCVI activities seen in the Tat/Env group provide a plausible mechanism responsible for the greater chronic-phase protection	(37)
2009	*M. mulatta*	Vaccination	Reduced acute viremia was significantly correlated with higher serum binding titer, stronger [ADCC] activity, and peak prechallenge and 2-week-postchallenge [ADCVI]	(38)
2009	*H. sapiens*	Infected patients	ADCC was detectable in all controllers tested and was significantly higher than in viremic individuals (*P* < 0.0002)	(39)
2005	*M. mulatta*	Vaccination	*In vitro* ADCC activity correlated with *in vivo* reduced acute viremia after a mucosal challenge with pathogenic SIV	(40)
2004	*H. sapiens*	Infected patients	Women with [cervical-lavage] ADCC activity had lower genital viral loads than did women with serum ADCC activity only	(41)
2001	*H. sapiens*	Infected patients	Serum titers of anti-HIV-1 ADCC antibodies bear a significant (*P* < 0.05) positive correlation with the peripheral blood CD+ T cell counts and a negative one with the number of copies of HIV-1 RNA in the plasma of HIV-infected individuals	(42)
2001	*H. sapiens*	Infected patients	ADCC effector cell function…correlated inversely with viral load (*R* = −0.42, *p* = 0.007) and directly with CD4^+^ cell counts (*R* = 0.52, *p* = 0.001)	(43)
			ADCC reduced virus yields from CD4+ lymphocytes infected with a primary HIV isolate	
2001	*H. sapiens*	Infected patients	The magnitude of this effector cell-mediated antiviral antibody response was inversely associated with plasma viremia level	(44)
1994	*H. sapiens*	Infected patients	Individuals with CD4 counts < 200/mm^3^ were found to have the lowest titres of [ADCC-mediating] antibodies in their sera	(45)
			ADCC-effector function of the [PBMCs] of HIV-infected individuals was significantly (*p* < 0.05) reduced as compared to PBMC from healthy, HIV-seronegative individuals	
1990	*H. sapiens*	Infected patients	Early ADCC responses were associated with high mean %CD4^+^ T cell numbers and absence of lymphadenopathy throughout the 2-year observation period	(46)
**Fc RECEPTOR-MEDIATED EFFECTOR FUNCTION IS ASSOCIATED WITH IMPROVED CLINICAL STATUS AND/OR DELAYED PROGRESSION TO (S)AIDS**				
2017	*H. sapiens*	Infected patients	[elite controllers] had higher levels of HIV Env-specific antibodies capable of binding Fc RIIIa, activating NK cells, and mediating granzyme B activity (all *P* < 0.01) than viremic subjects	(47)
2013	*H. sapiens*	Infected patients	Although the magnitude of ADCC responses in the LTSP cohort were not higher and did not correlate with CD4 T-cell depletion rates, the LTSP cohort had significantly broader ADCC responses compared with the non-LTSP cohort	(48)
2002	*M. mulatta*	Challenge model	Our study shows a correlation between humoral response, ADCC activity, and disease progression (as measured by CD4^+^ T cell counts)	(49)
			In these animals, ADCC activity is associated with delayed progression to AIDS	
1999	*H. sapiens*	Vertical transmission	Titres of [ADCC] were similar in transmitting vs. non-transmitting mothers… however, high [ADCC] titres were correlated with a good clinical status of children	(50)
1996	*H. sapiens*	Infected patients	Rapid progessors had significantly lower titres of Abs that mediate ADCC against HIV-1 gp120 than those of non-rapid progressors…or…non-progressors	(51)
			High titres of Abs that mediate ADCC correlate with successful host defense against AIDS	
1993	*H. sapiens*	Vertical transmission	Presence and titres of ADCC mediating and/or neutralizing antibodies in maternal sera did not predict HIV-1 infection in their respective children	(52)
			Significantly higher frequency of ADCC was seen in the seropositive non-AIDS children compared with the AIDS children	
1990	*H. sapiens*	Vertical transmission	ADCC antibody frequencies were much higher (70%) in the non-AIDS group than in the AIDS group (30%)	(53)
			HIV-specific ADCC and neutralizing antibodies do not seem to protect against transmission of HIV from mother to child but are significantly correlated with a better clinical stage of childhood HIV infection	
1988	*H. sapiens*	Infected patients	All sera of asymptomatic individuals… had a higher mean ADCC titer as compared to sera from patients progressing to AIDS or ARC	(54)
1987	*H. sapiens*	Infected patients	ADCC titers were lower in patients with [AIDS] than in asymptomatic carriers	(55)
1987	*H. sapiens*	Infected patients	Sera from healthy HTLV-III/LAV seropositive individuals in the presence of mononuclear cells from healthy HTLV-III/LAV seronegative donors exhibited significantly higher levels of ADCC activity compared to sera from patients with AIDS	(56)
**Fc RECEPTOR-MEDIATED EFFECTOR FUNCTION IS ASSOCIATED WITH A REDUCED RISK OF MORTALITY**				
2015	*H. sapiens*	Vertical transmission	ADCC levels were higher in uninfected than infected infants, although not significantly	(57)
			Increase in ADCC antibody activity in infected infants was associated with reduced mortality risk	
1999	*H. sapiens*	Infected patients	High baseline ADCC (>median) was associated with improved survival (*P* = 0.05)	(58)
**Fc RECEPTOR-MEDIATED EFFECTOR FUNCTION IS DISPENSIBLE TO PROTECTION AGAINST HIV-1/SHIV**				
2018	*M. mulatta*	Challenge model	The single V2 antibody at the dose given did not significantly reduce the number of infections	(29)
2018	*M. nemestrina*	Challenge model	The potent neutralizing capacity of PGT121 renders the Fc-dependent functions of the Ab at least partially redundant	(59)
2016	*M. mulatta*	Passive transfer	CH31 IgG1 and IgA2 isoforms infused before high-dose SHIV challenge were completely to partially protective, respectively, while [non-neutralizing]Abs (CH54 IgG1 and CH38 mIgA2) were non-protective	(60)
2016	*H. sapiens*	*Ex vivo* model	CD4 binding site bnAbs b12 IgG1 and CH31 IgG1 and IgA2 isoforms potently blocked HIV-1JR-CSF and HIV-1Bal26 infection. However, IgG1 and IgA nnAbs, either alone, or together, did not inhibit infection despite the presence of FcR-expressing effector cells in the tissue	(60)
2015	*M. mulatta*	Passive transfer	7B2 IgG1 or A32 IgG1, each containing mutations to enhance Fc function, was administered passively to rhesus macaques but afforded no protection against productive clinical infection	(32)
2014	*M. mulatta*	Passive transfer	Passive transfer of a low-dose of ADCC inducing antibodies did not protect from infection following SHIV-SF162P3 challenge	(61)
2013	*M. mulatta*	Challenge model	Despite virus-specific suppressive activity of the non-NAbs having been observed *in vitro*, their passive immunization post-infection did not result in SIV control *in vivo*	(62)
			Virion binding and ADCVI activity with lack of virus neutralizing activity were indicated to be insufficient for antibody-triggered non-sterile SIV control	
2014	*M. mulatta*	Vaccination	We identify blocking CD4+ T cell recruitment to thereby inhibit local expansion of infected founder populations as a second correlate of protection	(63)
			Virus-specific immune complex interactions with the inhibitory FcgRIIb receptor in the epithelium lining the cervix initiate expression of genes that block recruitment of target cells	
2012	*M. mulatta*	Passive transfer	NFb12 had higher affinity for human and rhesus macaque FcgR3a and was more efficient in inhibiting viral replication and more effective in killing HIV-infected cells in an ADCC assay	(64)
			Despite these more potent *in vitro* antiviral activities, NFb12 did not enhance protection *in vivo* against repeated low-dose vaginal challenge in the [SHIV/macaque] model compared to wild-type b12	
2011	*M. mulatta*	Challenge model	Compared with control animals, the protection by [neutralizing] b12 achieved statistical significance, whereas that caused by [non-neutralizing] F240 did not	(65)
2005	*M. mulatta*	Passive transfer	Six neonatal macaques were infused [subcutaneously] with immune IgG…positive for ADCC and Ab-dependent cell-mediated viral inhibition	(40)
			No protection, assessed by viral burdens, CD4 counts, and time to euthanasia was observed	
1994	*H. sapiens*	Vertical transmission	High levels of anti-HIV-1 ADCC antibody at birth are not protective against vertical transmission of HIV-1	(66)
1990	*H. sapiens*	Infected patients	There is no significant difference in ADCC values between those who remained asymptomatic and those who progressed to disease	(67)
1989	*H. sapiens*	Infected patients	Levels of serum and effector-cell ADCC activity do not predict whether an individual will develop AIDS	(68)

One of the most illustrative examples is the work of Hessell et al. which compared the protective efficacy of the broadly neutralizing antibody b12 with variants engineered to lack affinity for Fcγ receptors (FcγR) and/or the complement cascade-initiating protein C1q. Passive transfer of the native b12 was able to successfully block infection of rhesus macaques (27). Comparative analysis of the engineered variant lacking affinity for cellular antibody receptors to the crystallizable fragment (Fc) of IgG demonstrated that effector function driven by FcγRs, but not complement, contributed to protection. Similarly, humanized mouse models of HIV prevention and therapy have led to similar observations regarding the contribution of effector functions to the *in vitro* effect of other broadly neutralizing antibodies (69–71). However, given the lack of impact of effector function on the protection afforded by the neutralizing antibody PGT121 (59), and the inability of Fc glycosylation changes that result in enhanced ADCC activity specifically to enhance b12's protective efficacy (64), the generalizability of this observation to other antibodies, and the specific FcγR-dependent effector activities that may play a role in protection, remain to be determined. Nonetheless, a wealth of diverse data from studies of vaccines, monoclonal antibodies, and natural infection converge to point toward the importance of Fc receptor-mediated antiviral activities *in vivo*. Thus, fully leveraging the macaque model of HIV to identify vaccine and antibody candidates that bear desirable effector function profiles requires accounting for the ways in which immunoglobulins and their receptors differ between non-human primates and humans.

## Antibody Receptors

**Figure 1:**
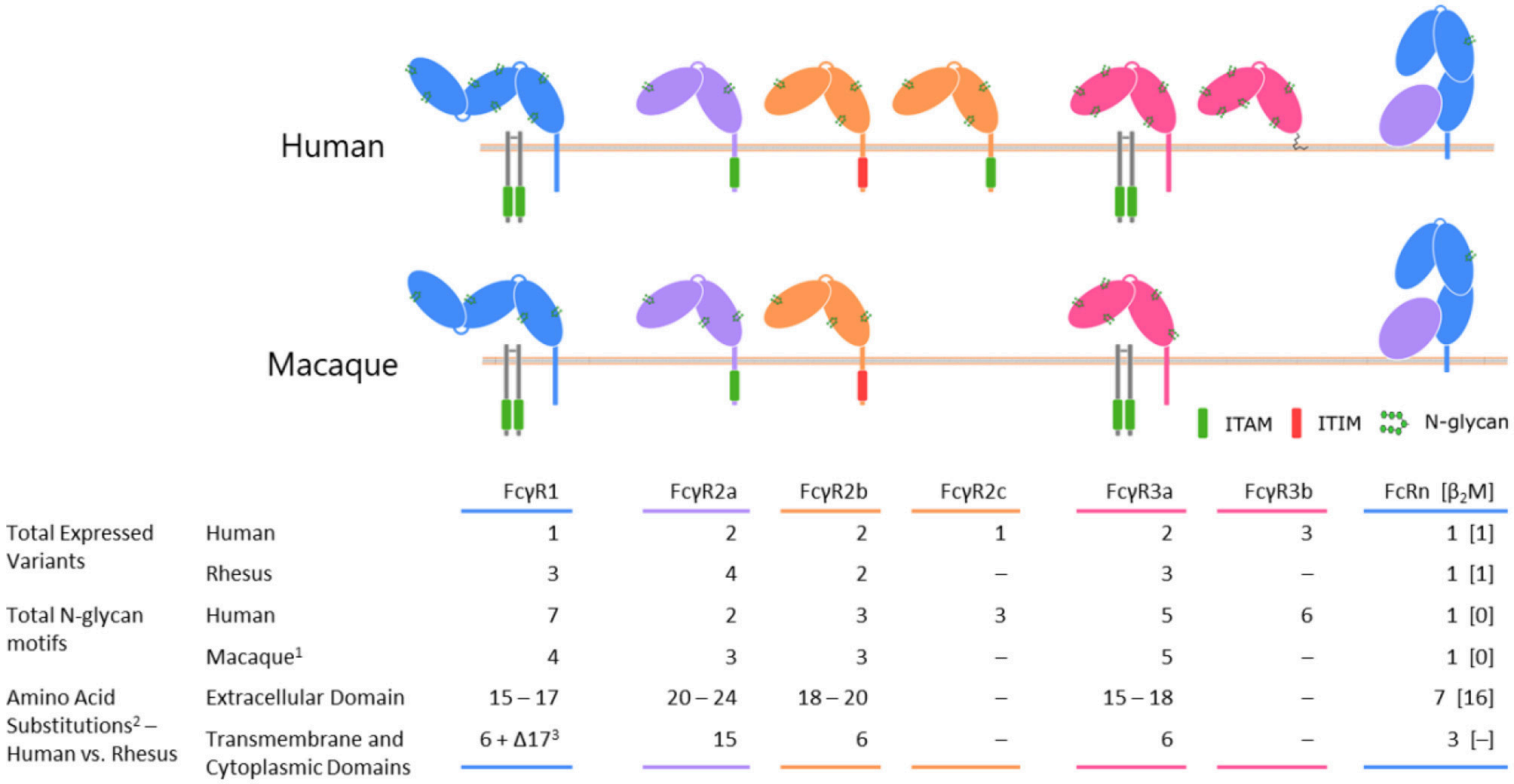
A comparison of selected Fc receptors between humans and rhesus macaque species. Differences in protein coding sequences reflects insertions, deletions, and sites of polymorphism. (1) The number of N glycan motifs is conserved across rhesus and cynomolgus macaques. (2) The ranges of values represent the minimum and maximum changes involved in shifting from human to rhesus; exact numbers depend on the allelic variants being compared. (3) Rhesus macaque FcγRI is truncated by 17 amino acids at its C terminus relative to the human variant.

The considerable interspecies diversity existing between the members of the FcγR family (itself a member of the immunoglobulin superfamily) (Figure 1) dramatically complicates comparisons of immune responses in humans and macaques. When cross-linked through their binding of antigen-antibody immune complexes, FcγRs transmit activating or inhibitory signals to the immune cell, depending on the identity of the signaling domain associated with the receptor in question. By enabling innate immune effector cells to potently respond to antigenic targets that the humoral immune system has adapted to recognize, FcγRs serve as a critical bridge between the arms of the immune system. Among other outcomes, FcγR engagement can lead to the release of cytokines, the direct killing of virus-infected, antibody-opsonized cells via ADCC, and phagocytosis and subsequent antigen processing and display (72).

This review will also briefly consider a receptor unrelated to the FcγR family—the MHC class I-like neonatal Fc receptor (FcRn). Much like the Fcγ receptors, FcRn acts as an important link between arms of the immune system, connecting humoral, and cellular immunity through its diverse activities. The receptor's capacity to distinguish monomeric IgG from that found in immune complex both imparts IgG with a lengthy serum half-life (73), and ensures that captured antigens are efficiently processes and presented to T cells (74, 75).

The differences in the sequences, ligand binding affinities, and expression patterns of human and macaque IgG and IgG receptors raise the possibility that a potent effector function-driven immune response may have a different phenotypic character in each species. This situation is further complicated by the frequent use of species-mismatched reagents and cell lines for *in vitro* activity assays. For example, the high baseline activity of macaque natural killer cells has driven most analyses of the ADCC potential found in the sera of vaccinated animals to be performed using human cell lines (40, 76). Similarly, the macaque/SHIV model is routinely used to evaluate the ability of passively transferred human antibodies to protect against infection (11–13). Absent a convincing case that the subclass of the transfused antibody or the receptor profile of the effector cell lines do not differ meaningfully between species, caution is required when attempting to extrapolate the findings of such studies from one species to another. Such risks may be mitigated, however, if given the means to more confidently translate observations between primate species and humans.

## Distinctions Between Human and NHP IgG Types

**Figure 2:**
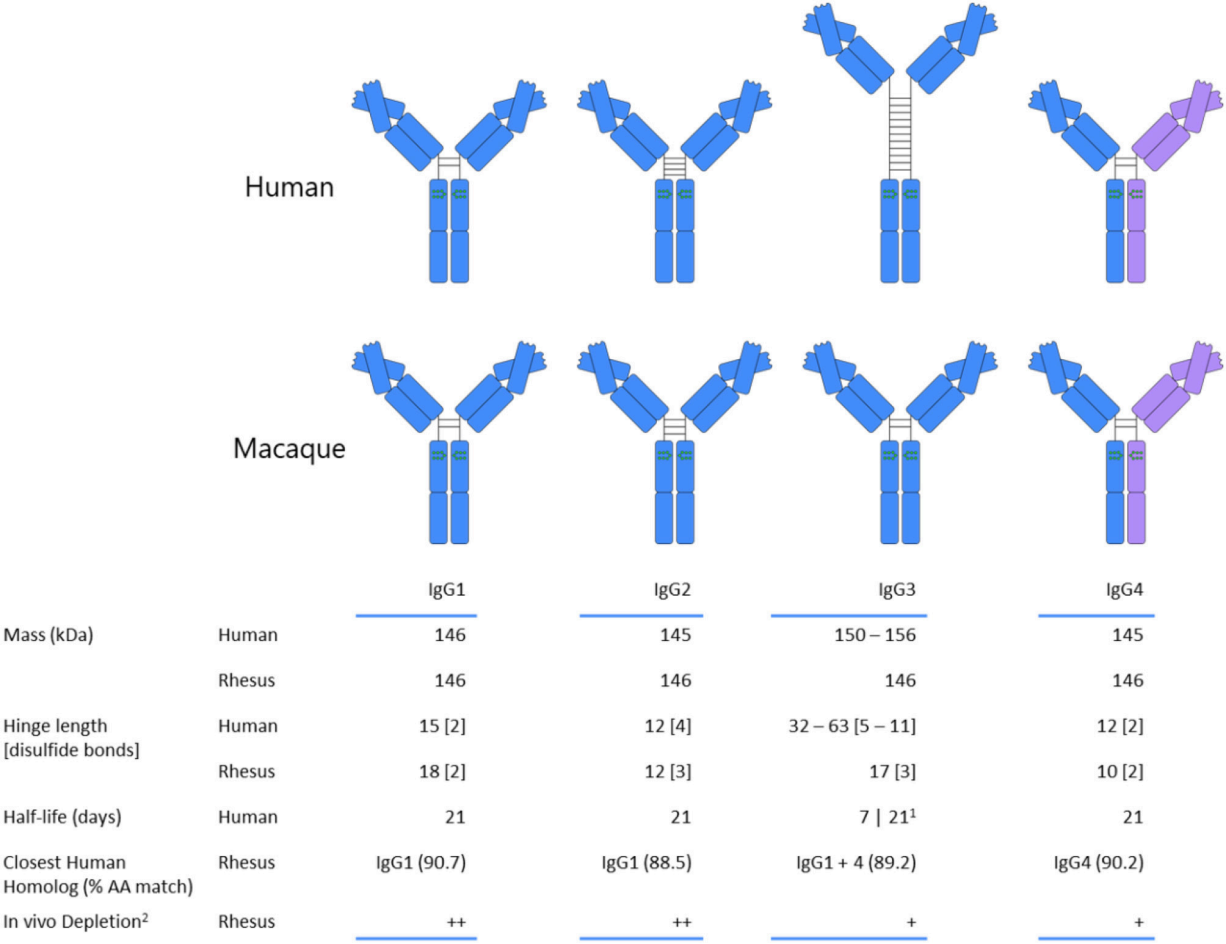
Comparison of human and rhesus macaque subclass properties. Human IgG3 is depicted with the most commonly expressed number of hinge unit repeats. IgG4 coloration reflects the potential for Fab arm exchange, which is a feature of human and rhesus IgG4, but not of cynomolgus macaques. References: (77–79). (1) The allotype-dependent inclusion or absence of His435 determines half-life of human IgG3, with histidine-containing having a longer half-life. (2) Plus signs denote the relative degree and durability of the decline in the CD8^+^ T cell population based on treatment with subclass-switched anti-CD8 monoclonal antibody (80).

The other participant in the FcγR-antibody interaction, immunoglobulin G, introduces an additional dimension that must be accounted for when translating observations between macaques and humans. Primate species nominally share the same IgG subclasses, having IgG1 through IgG4, numbered according to serum prevalence, but in the case of humans and macaques, the similarities largely end with this convention. Macaque IgG subclasses are in fact a much less structurally and functionally diverse group of proteins than those found in humans (Figure 2). For example, human IgG3 features an elongated hinge, up to 62 amino acids in length (81, 82), for which no analog has been found in any species of macaque. Human IgG2, on the other hand, has a shortened hinge, and exists in several disulfide bond-based structural isoforms whose activity profile can differ (83). The similarities between macaque subclasses do not end with their sequences, however; the functional activity of macaque IgG, assessed by the ability to promote monocyte phagocytosis and natural killer cell degranulation, and binding to FcγR is relatively consistent across each of the four subclasses in rhesus (80) and cynomologus (77), as compared to the more distinct activity and binding profiles observed for human IgG subclasses.

The human IgG response, conversely, can progress through better delineated phases during the course of infection, phases which may have arisen because they can collectively lead to an immune response activity profile that rises and falls when appropriate – fighting infection early on, and having the capacity to wane before the damage caused by inflammation is too severe. Within this framework, the role of IgG3, the first subclass within the immunoglobulin locus and the form that tends to be observed early on in infection (84), is to rapidly activate the immune response, particularly the effector cells of the innate immune system. In keeping with this role, human IgG3 strongly interacts with Fcγ receptors (85) and is a potent promoter of phagocytosis and degranulation. Switching downstream in the locus to IgG1 gives rise to this abundant, long-lived molecule. In fact, it has been theorized that the relative affinities of the human subclasses differ as beneficial mutations accumulate and switching to downstream subclasses occurs (86). By virtue of their generally reduced affinity for FcγR (85), subsequent or direct class-switching to IgG2 or IgG4 reduces activating potential, leading to the formation of less potent immune complexes (78). IgG4 may be particularly well-suited for this role because of its ability to undergo the process of Fab arm exchange, through which naturally-occurring bispecific antibodies are produced (87, 88). Because bivalent targeting normally aids in immune complex formation, the inability to avidly bind may further reduce the inflammatory potential of IgG4. Indeed, in humans, switching to IgG4 is often associated with amelioration of antibody-mediated allergy (89–92). Cynomolgus macaque antibodies cannot perform this Fab arm exchange (77, 93), and the quantity of IgG4 in macaques is thought to be quite minor (79, 80, 94).

Further, while the IgG subclasses are generally ordered from most to least activating in the immunoglobulin locus of humans (IgG3, IgG1, IgG2, IgG4), they are ordered from least to most activating in rhesus (IgG4, IgG3, IgG2, IgG1) (80, 95), though again, the differences in activity across rhesus and cynomolgus macaque subclasses are considerably less pronounced than in humans, and IgG sequences suggest this may hold in other macaque species as well (96). Left unanswered is the question of what significance observed polymorphisms in macaque IgG subclasses may hold—evidence of polymorphisms despite limited sequencing efforts may suggest the presence of significant allotypic diversity in rhesus and cynomolgus macaques (95, 97). It is likely that, to date, immunoglobulin sequence variation existing within macaque species has been undersampled.

While little is known about the progression of subclass switching over time in macaques, in humans, an early but waning IgG3 response has been observed in the setting of both natural infection and vaccination (16, 98, 99). Repetitive protein boosts in the setting of vaccination have also been observed to increase the magnitude of the IgG4 response (16, 17, 99). Most importantly, the range of effector activity differences and structural distinctions among subclasses are considerably more broad in humans than in macaques (80, 100). When making immunological comparisons between species, it is important to consider the ways in which human antibodies have been functionally honed that are not shared by the non-human primates relied on as research models.

## Distinctions Between Human and NHP FcγR

Members of the FcγR family share several common traits. Typically, two extracellular C-like domains are responsible for binding residues of IgG located in the lower hinge region (101–105). A molecule of IgG is bound by a single Fcγ receptor (106–108) through contacts made with both heavy chain constituents of the IgG homodimer. The character of those contacts differs between the protomers, with each side tending to favor a different class of intermolecular interactions (109). The extracellular domains of the receptors are followed by a transmembrane domain and a cytoplasmic domain, which may contain an activating or inhibitory signaling motif. In the absence of an intrinsic signaling motif, the task of communicating the antibody-mediated binding and receptor clustering is handled through association with the Fc receptor γ chain or other accessory receptors. An overview of the similarities and differences between receptors can be found in Figure 1.

Organization of this review will follow according to the type of signal a receptor transduces. Fcγ receptor crosslinking may lead to the generation of an activating signal though the involvement of an immunoreceptor tyrosine-based activating motif (ITAM) located in the cytoplasmic domain of the receptor or on the Fc receptor γ chain [reviewed in (72, 110–112)]. Briefly, activating receptors include the human and macaque FcγRI, FcγRIIa, and FcγRIIIa receptors. FcγRIIb, bearing an immunoreceptor tyrosine-based inhibitory motif (ITIM), is then the sole member of the inhibitory category of FcγR. The FcγRIIIb receptor found exclusively on human granulocytes, and apparently not present in the macaque genome, represents a special case as it lacks the normal transmembrane and cytoplasmic domains and is instead anchored to the cell membrane via a lipid chain. Although not as well-characterized as the other human Fcγ receptors, much of the available evidence suggests that FcγRIIIb contributes to the activation of granulocytes and that it is most appropriately included in the ITAM-bearing category of receptors.

### FcγRI

Among members of the Fcγ receptor family, FcγRI stands apart by virtue of its three extracellular domains and high ligand-binding affinity. As in humans, the receptor's elevated affinity in rhesus and cynomolgus macaques, has been measured in the low nanomolar to high picomolar range (77, 100), allows it to readily bind circulating monomeric IgG (85). As Figure 1 illustrates, the cytoplasmic domain of FcγRI lacks an intrinsic ITAM and this receptor instead utilizes the Fc receptor common γ chain to activate the cell (113, 114).

Characterization of FcγRI's role initially presented a challenge to researchers as the failure to express the receptor in humans apparently does not lead to any obvious immunodeficiency or associate with more frequent infections (115). Furthermore, the high concentration of IgG in circulation coupled with the receptor's high affinity suggested that FcγRI would be fully occupied by monomeric IgG at steady state (110). However, the conservation of this receptor across primate species and the rarity of individuals with expression defects suggests an important biological role. It is now thought that a primary role of FcγRI may be in antigen processing and display, as well as triggering the release of cytokines (116–118).

The single major allele of FcγRI expressed by humans (NP_000557) shares a high degree of amino acid sequence identity (~90%) with the macaque variants characterized to date, although several noteworthy differences do exist. The three major rhesus variants and one cynomolgus variant differ from the human receptor at four predicted contact residues and lack three N-linked glycosylation motifs found in the human molecule. Furthermore, in rhesus macaques (see NP_001244233), but not cynomolgus (NP_001270969), there is a 17 amino acid truncation at the intracellular C terminus. The consequences of this deletion, if any, for the receptor's association with the common γ chain have not been determined.

Within the macaque genus, FcγRI's extracellular domain phenotype is extremely similar, with the most common rhesus allele (FcγRI-1) and the major cynomolgus allele sharing identical extracellular domains. Furthermore, the rhesus FcγRI-2 allele differs from these at only two extracellular residues and the FcγRI-3 allele at just one. None of these polymorphic positions are predicted to contact the IgG Fc based on homology models. This high degree of conservation is also thought to extend to FcγRI's pattern of expression by immune cells. In keeping with its role in antigen presentation, the receptor is expressed by dendritic cells (117, 119), monocytes, and macrophages (120) (Table 2). Granulocytes are also capable of expressing FcγRI following induction by interferon γ (130, 131).

**Table 2:** Expression of members of Fcγ receptor family by immune cell subsets in humans and macaques.

**Lineage**	**Role**	**Cell type**	**Species**	**FcγRI**	**FcγRIIa**	**FcγRIIb**	**FcγRIIc**	**FcγRIIIa**	**FcγRIIIb**
Myeloid	Granulocytes	Neutrophils	*H. sapiens*	I	+	+/–	+	–	+
			*M. fascicularis*	ND	◦	◦	–	–	–
		Eosinophils	*H. sapiens*	ND	+	–	ND	–	+
			*M. fascicularis*	ND	◦	◦	–	–	–
		Basophils	*H. sapiens*	–	+	+	ND	–	+
			*M. fascicularis*	ND	◦	◦	–	–	–
		Mast Cells	*H. sapiens*	I	+	–	+/–	–	+
			*M. fascicularis*	ND	◦	◦	–	–	–
	Phagocytes	Monocytes/Macrophages	*H. sapiens*	I	+	+/–	–	+	–
			*M. mulatta*	+	◦	◦	–	+	–
		Dendritic Cells	*H. sapiens*	+	+	+	–	–	–
			*M. mulatta*	+	◦	◦	–	–	–
		Platelets	*H. sapiens*	ND	+	–	–	ND	ND
			*M. mulatta*	ND	ND	ND	–	ND	–
Lymphoid		B Cells	*H. sapiens*	–	–	+	+	–	–
			*M. mulatta*	–	◦	◦	–	–	–
		T Cells	*H. sapiens*	–	–	+/–^1^	–	+/–^2^	–
			*M. mulatta*	–	◦	◦	–	–	–
		Natural Killer Cells	*H. sapiens*	–	–	–	+	+	–
			*M. mulatta*	–	◦	◦	–	+	–

◦ Positive for FcγR2 but a/b not determined.

I Inducible by interferon γ.

ND Not determined.

+/– Expressed by a low percentage of cells, or by a specific subset.

Subset-specific expression is:

(1) *FcγRIIb has been observed on human memory CD8^+^ T cells (121)*.

(2) *FcγRIIIa has been observed on activated human CD4^+^ T cells (122)*.

*References: (72, 117, 119–135)*.

In light of the high degree of homology shared by human and macaque FcγRI, it should come as no great surprise that the receptors have strikingly similar preferences for IgG subclasses. Within a fully human context (receptor and IgG), FcγRI binds IgG1 and IgG3 with similarly high affinities, and has an intermediate preference for IgG4. The receptor demonstrates significantly reduced affinity for IgG2 (77, 85). In rhesus macaques, however, FcγRI has nearly equivalent affinities for all four subclasses of rhesus IgG (100); this trend is also observed in cynomolgus macaques (77). The cause of this difference is not the sequence of the receptors, but the IgG. This becomes apparent when mixing receptors and antibodies of different species—human FcγRI has relatively uniform affinity for different subclasses of macaque IgG while macaque receptors exhibit preferences between human subclasses (77, 100). Human IgG2 contains a unique motif (VAGP) in its upper CH2 domain, a motif not present in any macaque subclass or other human subclasses [instead—LLGGP, for all but human IgG4; accession numbers: [human] P01857; P01859; P01860; P08161 [rhesus macaque] see (79)], that impairs binding to FcγRI and accounts for this observation (136). Interestingly, human IgG4 has a slightly altered motif (FLGGP) at this position, which may explain the intermediate recognition of this subclass.

The high degree of FcγRI conservation between humans and macaques imposes fewer restrictions on the comparison process than is the case for other receptors handled in this review. IgG1, which is perhaps the most likely candidate for comparison due to its prevalence *in vivo* and among licensed therapeutics (137–139), is a particularly straightforward case and FcγRI-bearing macaque cells are expected to recognize a passively transfused human antibody near natively. In the case of human IgG2 and IgG4 however, there is evidence to suggest that while macaque FcγRI mirrors the human receptor's hierarchy of subclass preference outlined above, the receptor's affinity may be up to 4-fold stronger than in humans (77, 100). Since human IgG2 and IgG4 Fc domains have been used for monoclonal antibodies in instances where a silent Fc profile is desired (139), as well as for extending the half-life of therapeutic proteins by expression as Fc fusions, the heightened inflammatory potential of these molecules when evaluated in a macaque model may need to be taken into account.

### FcγRIIa

A sharp increase in complexity, both with and between species, occurs when surveying the most widely expressed activating receptor—FcγRIIa (140). This receptor plays a central role in the phagocytosis of antibody-opsonized antigens, which it triggers by means of an intrinsic ITAM located in its cytoplasmic domain (110).

Humans express two major allelic variants of FcγRIIa, which are distinguished by the identity of the amino acid found at position 131 of the extracellular domain (141). Expression of an arginine at this location results in weakened binding of IgG, particularly for IgG2 (142). Conversely, the histidine-containing variant demonstrates substantially higher affinity across multiple subclasses, including IgG2, and can effectively bind immune complexes composed of this subclass (85, 143). The consequences of this polymorphism can be dramatic. In the treatment of cancer, multiple monoclonal antibodies indicated for a range of malignancies have demonstrated differing responses based on the allelic makeup of the recipients (144). Protection against extracellular bacteria, often mediated by IgG2 targeting polysaccharide antigens, can also be impaired by the lower affinity allele; this may lead to an increase in the frequency and severity of certain infections among R131 individuals, particularly those homozygous for the allele (145–147). In the context of HIV, infected individuals that are homozygous for the lower affinity R131 variant experience a more rapid decline in CD4^+^ T cells than subjects having one or two copies of the high affinity H131 allele (148). Interestingly, no impact of this polymorphism on risk of infection was observed in the Vax004 HIV vaccine trial (149).

In macaques, a considerably greater number of major allelic variants have been characterized. Based on records in GenBank, numerous variants of this receptor have been defined in rhesus, pigtailed and cynomolgus macaques. While H131 is the minor allele in several human populations (150, 151), most macaque FcγRIIa alleles feature a histidine at this position and therefore resemble the high affinity human receptor in sequence and antibody recognition (100). The FcγRIIa-4 allele of rhesus macaques is an important exception to this trend, having a proline substitution at this residue that is likely detrimental to the receptor's affinity for IgG (100). Similarly, although the FcγRIIa-1 allele includes the critical histidine, it also contains an additional N-linked glycan motif at position 128, a residue that contacts the Fc region of IgG in the cocrystal structure of human proteins (152), which apparently interferes with the receptor's IgG affinity (100). The consequence of these differences is that alleles 1 (at least when produced recombinantly) and 4 have reduced affinity for macaque IgG compared to alleles 2 and 3 (100), creating high and low affinity variation analogous to the R131/H131 polymorphism in humans. Although the exact allelic frequencies have not been reported, the allotypic variants have been roughly numbered by prevalence (100). Accordingly, the FcγRIIa-1 allele is expected to occur most frequently, and its impact on passive transfer experiments and vaccine studies could prove to be substantial, provided that the native receptor is glycosylated similarly to the recombinantly-produced receptor utilized in *in vitro* studies. Relevant to the potential for differences between macaque and human FcγRII to drive differences in outcomes of antibody infusions between species, it is clear from evaluation of the binding profiles of engineered IgG sequence variants across human and rhesus receptors, that greater phenotypic differences are observed for FcγRII as compared to FcγRIII between species (153).

Receptor variation is not limited to the extracellular domain of FcγRIIa. While the four reported receptor alleles of rhesus macaques are identical intracellularly, they differ from the human and cynomolgus macaque variants in the sequence of their signaling domains. The activating ITAM motif takes the form of two semi-conserved sequences separated by six to eight amino acids. In the available sequences reviewed ([human] P12318; [rhesus] H9BMP0; [cynomolgus] Q8SPW4), the first ITAM unit takes the form Tyr-Met-Thr-Leu. Rhesus macaques repeat this sequence for the second iteration of the motif. Humans and cynomolgus macaques on the other hand use the slightly altered sequence of Tyr-Leu-Thr-Leu (154). This is in addition to a handful of residues flanking the second ITAM that are conserved among the available macaque sequences, but that differ when compared to the human receptors. The consequences of these substitutions, if any, are not known.

The comparison of sequence diversity alone is not sufficient to capture all of the differences between human and macaque FcγRIIa; among other factors, the patterns of expression must also be accounted for when translating observations between species. In humans, FcγRIIa is the most commonly expressed Fc receptor of monocytes and macrophages (155), and is also expressed by dendritic cells and granulocytes. Analysis of expression in macaques has been complicated by the fact that the extracellular domain of FcγRIIa and the inhibitory FcγRIIb receptor have even more highly conserved amino acid sequences (95%) than is the case in humans (92%) (100) ([human] P12318 vs. P31994; [rhesus] H9BMP0 vs. F7GVR0; [cynomolgus] Q8SPW4 vs. Q8SPW3). Difficulty in obtaining flow cytometry reagents capable of reliably discriminating between the receptors has meant that much of the literature published to date omits the specific form of the receptor expressed by a given cell type, reporting instead only the presence or absence of FcγRII (156). Even so, there is data that suggests that cynomolgus and pigtail macaque neutrophils express 3–5 fold more total FcγRII than their human counterparts (77, 156), which are known to express only the activating form of the receptor (132). Given this known difference in expression levels and additional differences potentially associated with activating vs. inhibitory and allotypic FcγRII composition, there is the potential for altered neutrophil biology in non-human primate model systems.

### FcγRIII

In many of the studies summarized in Table 1, subjects producing antibodies capable of carrying out directed killing of virally-infected cells through the process of antibody-dependent cell-mediated cytotoxicity (ADCC) experienced significantly better clinical outcomes. In macaques and most, but not all, humans, the cell type most commonly associated with performing ADCC, natural killer (NK) cells, exclusively express FcγRIIIa, making the receptor a potent potential contributor to antiviral effector function. Humans express two major allelic variants of FcγRIIIa that are distinguished based on differing levels of activity attributed to a single polymorphic residue in the extracellular domain—position 158 in this case (157, 158). Expression of a valine at this position results in greater affinity for human IgG1 and IgG3, and to a lesser extent IgG4, as compared to the phenylalanine-containing variant (85). Although it is found less frequently in the population (159), the V158 allele can confer significant benefits. For example, V158 individuals respond more strongly to anti-tumor monoclonal antibody therapy, an observation attributed to enhanced ADCC directed against the tumor cells (160–162).

The extracellular domain of rhesus macaque FcγRIIIa also exhibits a polymorphism at position 158. However, the isoleucine expressed by the FcγRIIIa-1 and−2 alleles has similar side chain chemistry to the valine of FcγRIIIa-3 and as a result, the extracellular domains of known rhesus alleles resemble the high affinity human receptor in both sequence and affinity (153). Interestingly, the FcγRIIIa-2 allele, which is identical to FcγRIIIa-1 extracellularly but carries a pair of polymorphisms that introduce valine residues to the transmembrane domain and membrane-proximal region of the cytoplasmic domain (positions 229 and 233, respectively), is associated with more complete depletion of B cells following treatment with rituximab (163). More broadly, the amino acid sequences of the non-human primates surveyed maintained > 90% homology to humans in their extracellular domains. In each of the macaque sequences though, there is an altered residue that leads to the elimination of a N-glycan motif known to be modified in humans. This is potentially offset by the introduction of a new motif close by, but it is not known whether this new site is accessible for glycosylation.

Based on biophysical and functional data gathered thus far (77, 100, 153), one can reasonably conclude that the macaque forms of the receptor are a fair approximation of the high affinity human allele, FcγRIIIa V158. Unfortunately, the prevalence of the lower affinity F158 allele in humans exceeds that of the high affinity allele (164–166). All else being equal, this difference in allelic frequencies suggests that most humans may be likely to generate a less robust ADCC response than a macaque would based on the lower affinity of their FcγRIIIa receptor. Significantly, human polymorphic variation at this position has been associated with rate of infection among low risk HIV vaccine recipients, and development of Kaposi's sarcoma, an AIDS defining illness, in HIV infected men (148, 167).

Human granulocytes uniquely express a variant of FcγRIII that is apparently not found in any species of macaque—FcγRIIIb (168). The receptor's homology to the extracellular domain of human FcγRIIIa and the weight of experimental evidence generated to date argue for the inclusion of FcγRIIIb alongside the activating Fcγ receptors. This classification has been far from straightforward however (169), in part because FcγRIIIb uniquely lacks both a transmembrane domain and cytoplasmic signaling domains, instead being tethered to the cell membrane via a glycosylphosphatidylinositol (GPI) anchor (168). Even so, upregulation of FcγRIIIb in response to engagement has been observed as well as an association with Syk signaling in neutrophils (170). In order to transduce such signals, FcγRIIIb would require an accessory signaling element, the proposed candidates for which include the complement receptor (171) and FcγRIIa (172). The latter receptor has been hypothesized to act in synergy with FcγRIIIb to increase the flux of Ca^2+^ into granulocytes and enhance their effector functions (173), which include the release of cytotoxic granules (174) and phagocytosis (175, 176). Conversely, a competing view of FcγRIIIb holds that the receptor merely serves as a decoy that competes with activating receptors for immune complexes. Experimental support for this model lies in the observation that enhancing the affinity of IgG for FcγRIII broadly resulted in lower levels of neutrophil ADCC (177). This alternate hypothesis raises the possibility that human granulocytes may have a means to receive an inhibitory input that is not available to macaque effector cells, potentially further skewing signaling in neutrophils.

## Inhibitory Receptor

### FcγRIIb

All humans and macaques express a single form of inhibitory FcγR—FcγRIIb. The receptor contains an intrinsic ITIM in its cytoplasmic domain that allows it to rein in immune responses. FcγRIIb is abundantly expressed by phagocytic cells (120, 132) and is typically the sole Fcγ receptor found on human B cells (132, 133). It is additionally expressed by liver sinusoidal endothelial cells, where it is thought to play an role in recognition and uptake of immune complexes (178, 179). Evaluation of the role of this receptor on immune cells has been especially hampered by the limited availability of staining reagents capable of discriminating FcγRIIa from FcγRIIb. In humans, an FcγRIIb-specific reagent is available (132), however, its specificity toward macaque receptors has not been reported. All major human variants of FcγRIIb feature identical extracellular domains and contain an arginine at residue 131 (P31994), making them phenotypically more similar to the low affinity variant of the activating FcγRIIa (85, 153). Of the two human alleles which have been characterized, the rarer variant contains an isoleucine to threonine mutation in the transmembrane domain, at residue 232. This change renders the receptor incompatible with inclusion in lipid rafts and as a result, unable to signal (180, 181).

In contrast to humans, both reported alleles of rhesus macaque FcγRIIb feature a histidine at position 131 and therefore more closely resemble the high affinity variants of FcγRIIa. This is superceded, however, in the FcγRIIb-2 allele by a leucine to proline substitution at residue 88, a site immediately adjacent to a predicted Fc contact residue, which abrogates the receptor's affinity for all subclasses of both rhesus macaque and human IgG (100). While this variant is the less common of the two alleles, its precise frequency has yet to be reported. However, it is possible that the inclusion of FcγRIIb-2 animals in a study could have significant consequences. Intracellularly, FcγRIIb is well-conserved between humans and macaques; the cytoplasmic domains differ at just two residues ([human] P31994; [rhesus] F7GVR0; [cynomolgus] Q8SPW3). Of these differences, one is a conservative change from isoleucine in humans to valine in rhesus macaques. Furthermore, neither of the mutations occurs proximal to the signaling motif.

That FcγRIIb is the sole inhibitory Fc receptor in humans and macaques means the consequences of the interspecies FcγRIIb differences have the potential to be far-reaching. For example, according to one proposed model of SIV and HIV infection, FcγRIIb engagement at the mucosa somewhat paradoxically has been suggested to contribute to protection by generating an inhibitory signal that slows the migration of CD4^+^ T cells to the region and deprives the virus of host cells for replication (63). However, the high affinity phenotype of macaque FcγRIIb could mean that the receptor stands on an equal footing (or greater, in the case of FcγRIIa alleles 1 and 4) with the activating receptors in the competition for immune complexes—a scenario that is not recapitulated in humans where even FcγRIIa-R131 has higher affinity for IgG than FcγRIIb (85, 100).

## Additional Receptors

### FcγRIIc

In ~20–45% of humans (182, 183), an unequal genetic crossover between the activating cytoplasmic domain of FcγRIIa and the extracellular domain of FcγRIIb results in the expression of the receptor FcγRIIc (184) on B cells and natural killer cells (134, 135). FcγRIIc provides an additional means of activating immune cells and is capable of triggering ADCC in natural killer cells (185). In the context of HIV infection, the receptor is noteworthy based on the observation that a single nucleotide polymorphism located in an intron of FcγRIIc was associated with greater vaccine efficacy in the RV144 trial (186). A similar receptor has not been reported in macaques.

### Neonatal Fc Receptor (FcRn)

The similarities between macaques and humans that make these non-human primates a tractable model for the study of HIV also make them useful for determining the pharmacokinetic properties of biologic drugs. Cynomolgus macaques in particular are a widely utilized species for the evaluation of antibodies and Fc fusion proteins (187), biologics that depend heavily on FcRn to achieve half-lives that can extend beyond 3 weeks (73, 188–191).

FcRn is a heterodimeric protein composed of a β_2_-microglobulin (β_2_m) subunit and an MHC class I-like heavy chain (192–194). Diverse tissues and cell types express FcRn (195–198), but it functions principally in intestinal epithelia, vascular endothelia, and syncytiotrophoblasts (199–203) where the receptor's ability to bind to the Fc portion of IgG in a pH-dependent manner, having high affinity under acidic conditions (pH < 6.5) and relatively low affinity at a physiological pH of 7.4 (204–206), permits the uptake, transfer, and release of IgG across compartments of the body. This property of FcRn enables the maternofetal transfer of IgG across the placenta (207) and the absorption of IgG present in maternal milk by the intestinal epithelium of neonatal rodents (204, 208). FcRn's high level of expression in vascular endothelial cells leads to the sorting and recycling of endocytosed IgG from acidified endosomes, where the receptor's affinity for IgG is high, to circulation, where the physiological pH causes the release of IgG (73, 189–191). Additionally, FcRn can contribute to the accelerated processing of antigens derived from multivalent immune complexes and their subsequent presentation by both forms of major histocompatibility complex (74, 75).

The heavy chain of FcRn is well-conserved among the macaque species commonly used in biomedical research; the most frequent variants are identical to one another (accession numbers: NM_001257520, NM_001284551, and XM_011768102). Among the ten residues that differ between humans and macaques, none occur in a location known to contact the Fc, or in the receptor's N-linked glycan sequon (209). Similarly, of the four polymorphisms observed in cynomolgus macaques, only two occur in the extracellular domain and neither involves putative IgG Fc contact residues (210). Interestingly, a promoter polymorphism in FcRn has been identified to associate with reduced levels of serum IgG, though the impact of this polymorphism on the half-life or biodistribution of passively administered mAb or polyclonal samples remains to be defined (211).

The β_2_-microglobulin subunit that makes up the remainder of the heterodimeric receptor is somewhat more diverse than the heavy chain, both between species of macaques and when macaques are compared to humans; the N-terminal isoleucine required for interaction with IgG (212) is present in all species under review, and the impact of sequence diversity in this protein has been less well-studied in all species.

## Conclusions

The sheer number of factors to consider when translating observations between macaques and humans makes the process a challenging, multidimensional one. Differences in the structures and activities of IgG subclasses, and polymorphisms in protein sequence and post-translational modification of antibody receptors are a subset of the many relevant considerations. Copy number variation, splice variants, and alleles with sequence variation outside of coding regions have been associated with a diversity of phenotypes in humans (183, 213–217), and are presumed to exist in NHP. A number of differences in the patterns of cellular expression of FcγRs between species are well-established, and while genetically-associated differences in expression levels likely exist, they have not been fully characterized. Linkage disequilibrium between FcRs and a diversity of major and minor alleles make it difficult to define the potential effect of these polymorphisms with high confidence even in large cohorts. Thus, the process of precisely translating results between species is a complex and potentially intractable challenge. However, the highly significant contributions to drug development demonstrates that macaques can nonetheless serve as an excellent preclinical model.

When considering exceptional scenarios, several simplifying assumptions may help to smooth the process. The first—treating the members of the macaque genus largely interchangeably—has already achieved widespread adoption (156) as the shifting landscape of cost, conservation, and ethical considerations have driven changes in the decades since the model's introduction in the preferred macaque species, and among NHP more generally, for biomedical research (218). In light of the considerable similarities between macaque species (including shared allotypic diversity) outlined in this review, it is our view that under common research circumstances, this practice serves as a useful simplifying assumption; however, exceptions do exist. For example, evaluation of passive infusion of human IgG4 in NHP is one such example. Human IgG4 can undergo Fab arm exchange with endogenous human IgG4, but would not exchange with any serum antibody in some, though not all macaque species. Similarly, outcomes of vaccination studies in humans have been associated with the subclasses of antibodies induced. However, the extensive differences between the repertoire of subclasses in humans and the relatively more monolithic structural and activity profiles of NHP IgG types may pose challenges to easy translation of vaccine studies. These differences may preclude attempts to model means to induce specific subclasses by altered regimens, adjuvants, and immunogens, or to explore the relevance of class-switching patterns observed in humans to challenge outcomes.

Of the cell types and FcγR-mediated immune processes potentially impacted by the differences in human and macaque biology, two in particular stand out as deserving of additional comment. Unlike the low affinity phenotype of human FcγRIIb, the inclusion of a histidine at a critical contact residue in macaques grants equivalent affinity (or greater, in the case of alleles 1 and 4) to that of the activating FcγRIIa. While this has the potential to alter the ratio of activating and inhibitory signals relative to what is observed in humans for any cell type expressing a mix of receptors, the effect could be even more pronounced in B cells where FcγRIIb is the only member of the family expressed.

Neutrophil biology is another aspect of macaque immunity likely to differ significantly from that of humans based on differences in Fcγ receptors. The reasons for this are twofold. The first is the substantially higher level of expression of FcγRII receptor that has been observed on macaque neutrophils. This expression profile potentially contributes to a stronger response by these cells in macaques, although this assumes that macaque neutrophils, like those of humans, express FcγRIIa, and do not express inhibitory the FcγRIIb, which could offset the larger number of activating receptors. The second major difference is the unique expression of FcγRIIIb, which is absent from macaques, by human neutrophils, although the effect of expression of this receptor is more difficult to determine as its role has not been definitively determined.

The recent history of biomedical research well-illustrates the utility of the members of the macaque genus for modeling human diseases and evaluating therapeutic interventions. Much of the progress left to be made in understanding the non-human primate model resides on the margins and need only be considered under particular circumstances. However, consideration of the interspecies variability that has been defined to date and the gaps in our present understanding, if filled, have the potential to make macaques an even more enabling model organism. These insights in turn may increase the rate at which pre-clinical observations are converted to clinical success stories.

## Author Contributions

All authors listed have made a substantial, direct and intellectual contribution to the work, and approved it for publication.

### Conflict of Interest Statement

The authors declare that the research was conducted in the absence of any commercial or financial relationships that could be construed as a potential conflict of interest. The reviewer AC declared a past co-authorship with one of the authors MA to the handling editor.
